# Artificial neural network in predicting bladder cancer recurrence

**DOI:** 10.1186/1897-4287-10-S1-A3

**Published:** 2012-01-12

**Authors:** Edyta Borkowska, Maria Constantinou, Adam Jędrzejczyk, Magdalena Traczyk, Monika Banaszkiewicz, Michał Pietrusiński, Piotr Marks, Marek Rożniecki, Andrzej Kruk, Bogdan Kałużewski

**Affiliations:** 1 Department of Clinical Genetics, Medical University of Lodz, Poland; 2 Department of Urology, Medical University of Lodz, , Poland; 3 Urologists “Marek Rożniecki and Partners”, Lask, Poland; 4 Department of Ecology and Vertebrate Zoology, University of Lodz, Poland

## Introduction

The more we learn about human genetics and human variation, the more apparent it becomes that our individual make-up has a noticeble impact on the effectiveness of medications. Urinary bladder cancer is the sixth leading cause of mortality due to malignant neoplasm among Polish man in Lodz region. Many genetic and epigenetic alterations have been identified that contribute directly to the development of bladder tumors. The aim of the project was the creation of the individual risk calculator of bladder cancer recurrence using available clinical data and the results of the long term genetic research.

## Material and methods

Experimental molecular markers (including P53 and CDKN2a mutations and polymorphisms, assessment the level of genes expression, loss of heterozygosity), UroVysion test results, HPV infection status and conventional clinicopathological data were studied in a cohort of 104 patients (92 men) with bladder cancer diagnosed between November 2006 and June 2008 (the mean age was 67). 44 lesions were determined to be non-invasive tumors (pTa), whereas 36 were invasive (pT1-T4). Tumor grade was noted low (G1) in 46 cases and high (G2-3) in 34 cases. For modeling the relationship between the explained variable (time whithout recurrence) and explaining variables a multilayer perceptron was used (a kind of artificial neural network). Its functioning can be compared to how the so called "black box". We know what data are introduced, we are aquinted with the obtained result, however we have no knowledge of what process takes place inside.

## Results

The predicted variable was coded as follows: 0-high risk of recurrence and death, 1- early recurrence, 2-late recurrence, 3- very low risk for recurrence. Next, the set of data was divided into three sub-groups: training (65 cases), validation set (20 cases) and test set (19 cases). The fact that artificial neural networks create models of phenomena only on the basis of representative data previously gathered by investigators is useful in several aspects. Firstly, for using artificial neural networks a priori knowledge of the problem in the statistical sense is not required. Secondly, the construction of the model may result in identification of new important variables, which possibly would be ignored using conventional statistical analysis. Thirdly, it should be noted that the artificial neural networks are able to deal with lack of data, the problem which often encountered in clinical practice.

## Conclusion

The calculator proposed by us takes into considerations clinicopathological, genetic and environmental data (like tobacco smoking and occupational exposure). To our knowledge this is first attempt to include a various genetic variable into the bladder cancer recurrence calculator. We intend to increase the amount of data presented to the net so the process of net training can be more effective and it can allow calculating the risk of disease progression. In our opinion this is the direction which should be followed, especially in the oncoming era of personalized healthcare and individual therapy.

